# Characterisation of Environmental Biofilms Colonising Wall Paintings of the Fornelle Cave in the Archaeological Site of Cales

**DOI:** 10.3390/ijerph18158048

**Published:** 2021-07-29

**Authors:** Daniele De Luca, Paolo Caputo, Teresa Perfetto, Paola Cennamo

**Affiliations:** 1 Department of Biology, University of Naples Federico II, Via Foria 223, 80139 Naples, Italy; daniele.deluca@unina.it (D.D.L.); paolo.caputo@unina.it (P.C.); 2 Department of Humanities, University of Naples Suor Orsola Benincasa, Via Santa Caterina da Siena 37, 80132 Naples, Italy; teresa.perfetto@docenti.unisob.na.it

**Keywords:** ARISA, biofilms, microbial diversity, microscopy, votive caves

## Abstract

Caves present unique habitats for the development of microbial communities due to their peculiar environmental conditions. In caves decorated with frescoes, the characterization of microbial biofilm is important to better preserve and safeguard such artworks. This study aims to investigate the microbial communities present in the Fornelle Cave (Calvi Risorta, Caserta, Italy) and their correlation with environmental parameters. The cave walls and the wall paintings have been altered by environmental conditions and microbial activity. We first used light microscopy and scanning electron microscopy (SEM) and X-ray diffraction to characterise the biofilm structure and the mineral composition of substrata, respectively. Then, using both culture-dependent (Sanger sequencing) and culture-independent (automated ribosomal intergenic spacer analysis, ARISA) molecular methods, we demonstrated that the taxonomic composition of biofilms was different across the three substrata analysed and, in some cases, positively correlated with some environmental parameters. We identified 47 taxa in the biofilm samples, specifically 8 bacterial, 18 cyanobacterial, 14 algal and 7 fungal taxa. Fungi showed the highest number of ARISA types on the tuff rock, while autotrophic organisms (cyanobacteria and algae) on the frescoes exposed to light. This study confirms that caves constitute a biodiversity-rich environment for microbial taxa and that, in the presence of wall paintings, taxonomic characterization is particularly important for conservation and restoration purposes.

## 1. Introduction

Caves and other hypogeal habitats are extreme environments where high concentrations of minerals and the oligotrophic conditions caused by the scarcity of light and primary producers allow the survival of mostly extremophile microorganisms [1,2]. Cave microbiota are composed of metabolically diverse organisms, such as autotrophs, heterotrophs and mixotrophs. Most autotrophic microorganisms get their energy through chemosynthesis (chemoautotrophs), using chemical elements in the rocks (e.g., bacteria); a small fraction (algae and cyanobacteria) uses photosynthesis (photoautotrophs). Heterotrophic microorganisms feed on decaying organic matter (e.g., plant debris, guano and carrion) [3,4], while mixotrophs utilise both heterotrophic and autotrophic means. Chemoautotrophs, heterotrophs and mixotrophs generally occur in all the parts of a cave, even the most internal, while photoautotrophs are limited to the outermost parts of the caves where the photosynthetically active radiation (PAR) is available [5,6]. However, dimly lit caves can also have diverse photosynthetic communities [7,8]. Cave microbiota generally include various taxonomic units: bacteria, actinobacteria, archaea and fungi and, more infrequently, microalgae and cyanobacteria [2]. Among microalgae, the most important groups are Chlorophyceae and Bacillariophyceae (diatoms), of which little is known in comparison to their marine and freshwater counterparts [4].

Generally, epilithic cyanobacteria and green algae are the first colonizers of illuminated cave walls [9], with cyanobacteria playing a key role in the genesis of biofilms through the production of exopolymeric substances (EPS) that allow the adhesion to rocks [10]. Organic matter produced by autotrophs is then utilised as energy source by heterotrophs (fungi and other bacteria), which eventually leads to the establishment of a microbial community. The colonisation of the substrate (bare rock, cave paintings) can cause changes in its chemical and physical properties, i.e., biodegradation. However, the invasion of materials by living organisms does not necessarily lead to physical and chemical degradation but, sometimes, simply to reversible colour changes [11]. Therefore, the term “bioreceptivity” [11] was introduced to indicate the aptitude of a material to be colonized by microorganisms without necessarily undergoing biodeterioration. The proliferation of microorganisms on substrata leads to the formation of a layer of white, grey or green patinas due to the formation of the biofilm.

Several studies have focused their attention on the microbial composition of biofilms on stone monuments. Although the vast majority have described biodeterioration problems on stone monuments or mortar structures and murals in the last few years, several show caves were investigated in detail. These include the Altamira Cave [12,13], Tito Bustillo [14,15], La Garma and Llonin [16] caves in northern Spain and Grotta dei Cervi in Porto Badisco, Italy [17].

This study aims to assess the microbial community composition of biofilms growing in the votive cave of Fornelle in the Calvi Risorta archaeological site (Campania Region, Italy) and its correlation to environmental variables. The cave hosts numerous frescoes dating back to the middle ages. Most of the paintings are covered with a visible layer of calcium carbonate, which acted as a protective layer for the wall paintings, but has facilitated abundant colonization by biodeteriogenic microorganisms (together with the surface layer of synthetic resin covering some frescoes).

## 2. Materials and Methods

### 2.1. Study Area

**Figure 1:**
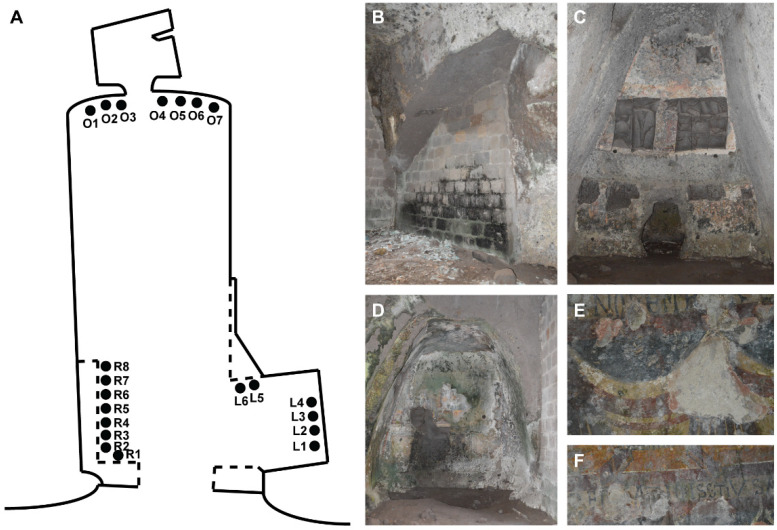
The Fornelle cave in Calvi Risorta. (**A**) Schematic representation of the cave (redrawn from [24]), indicating the sampling points (R = tuff rock; L = light fresco; O = dark fresco); (**B**) left side of the cave; (**C**) end wall of the cave; (**D**) end wall of the chapel (right side of the cave); (**E**) and (**F**) details of the frescoes on the left side of the chapel.

The Fornelle cave is located in the South-eastern area of the ancient Cales (now Calvi Risorta, Campania, Italy: 41°11′50.51″ N, 14°8′7.89″ E), a city that was a crossroads of great ancient civilizations: the Aurunca, the Etruscan, the Latin, the Samnite [18,19]. The tuff cave, likely man-made and related to tuff-quarrying activities [20] became initially a public cistern, and was transformed into a church in medieval time with the creation of some frescoes [21,22]. It is composed of three rooms (Figure 1A): a large, rectangular basin (5.7 × 14.8 m) with a trapezoidal section, a small room (about 2.3 × 3 m) at the end of the former, and a third room, the chapel (2.5 × 3.6 m), with a quadrangular plan and located to the right of the entrance to the main basin [23]. The left walls of the Fornelle cave bears highly damaged frescoes with scenes from the Banquet of Herod and the Beheading of John the Baptist [24], as well as biofilm-colonised bare rocks (Figure 1B). On the end wall of the cave is the panel of the Ascension, whose frescoes were stolen (dark grey panels, Figure 1C), but later recovered and exhibited at the Museum of the Opera and the Territory at the Royal Palace of Caserta. An inscription shows the names of Count Pandolfus and his wife Gualferada, who commissioned the frescoes between the second half of the 11th century and the beginning of the 12th century [24]. On the right side of the cave there is a chapel: its end wall bears a damaged panel with a votive scene (Figure 1D), while on the left side a fresco can be observed with a Latin inscription indicating the day and month of construction of the altar (1 November, see Figure 1E,F). These frescoes were commissioned a few decades later (last quarter of the 11th century–first quarter of the 12th century) by an Icmundus and his family [24].

### 2.2. Sampling Points

Twenty-one samples of biofilm were collected from the cave (Figure 1A) in the autumn of 2018 and labelled according to the position and the characteristics of the substratum as follows: the left side as “tuff rock” (R1–R8), the end wall as “dark fresco” (O1–O7) and the right side (chapel) as “light fresco” (L1–L6). At the time of sampling (September 2018), the monthly minimum and maximum temperatures of the area were 17.2 °C and 28.6 °C, respectively; and min and max relative humidity were 38% and 86%, respectively (data recorded by the meteorologic station of Grazzanise, Caserta). Each specimen was taken by scraping the substrate with a sterile scalpel and putting the sample into a sterile tube. For each sampling point, we recorded the following environmental parameters: temperature (°C), light intensity (lx) and relative humidity (%) (Table 1). The measurements of temperature and relative humidity were carried out using the TESTO 177 H-1 data logger (Testo Middle East FZCO, Dubai, United Arab Emirates), while light intensity measurements were made using the TESTO 545 digital light meter.

### 2.3. Light and Scanning Electron Microscopy Analyses

Light microscopy and scanning electron microscopy (SEM) observations were conducted on all the biofilm samples to identify the algal, fugal and microbial components and to examine the microbial organization in the biofilms. Optical observations were conducted on multiple samples to examine the relationship between the biofilm and the substratum. For the taxonomic identification of cyanobacteria we followed [25], for green algae [26]. Light microscopy analyses were conducted using a Nikon Eclipse L150 (Nikon, Tokyo, Japan) optical microscopy. Micro-samples of artificial biofilm were directly observed at SEM. A Tescan Vega 3 scanning electron microscope with EDS microanalysis (with a lanthanum hexaboride, LaB6, electron source) was utilized. It was equipped with a LaB6 filament with best resolution of 2 nm at 30 kV in high-vacuum mode and 2.5 nm at 30 kV in low-vacuum mode. It also had a panchromatic CL detector with 185–850 nm wavelength range, a low-vacuum SE detector, transmitted electron detector and IR TV camera for chamber viewing.

### 2.4. X-ray Diffraction (XRD) Analysis

The mineralogical phases of substrata colonised by biofilms were analysed through X-ray diffraction for a qualitative and semi-quantitative determination of the components. We took one sample from the bare tuff rock on the left side of the cave (RS), one from the end wall (OS), and another one from the left side of the chapel (LS). Samples were ground and finely pulverized before the analysis, which was carried out with a Miniflex Rigaku X-ray diffractometer (Rigaku Americas Holding Company, The Woodlands, TX, USA) with cobalt tube operating at 30 KV and 15 mA and counting time set as 3600 s.

### 2.5. Molecular Analyses

The bacterial, algal and fungal communities inhabiting the Fornelle cave were determined as follows: (1) analysis of dominant taxa occurring on tuff, dark fresco and light fresco biofilm samples using culture-dependent approaches and Sanger sequencing; (2) characterisation of total community in each sampling point using the automated ribosomal intergenic spacer analysis (ARISA) technique.

#### 2.5.1. Culture-Dependent Characterisation of Biological Community

A small fraction of each biofilm sample was put in culture in Petri dishes containing selective media for algae (Bold basal medium, BBM, [27]) and cyanobacteria (BG-11, [28]). For the other microorganisms, we used specific media for either bacteria [29] or microfungi (e.g., potato dextrose agar, PDA).

Total DNA was extracted from each culture in the Petri dishes following the procedure described by [30]. The 16S and 18S rRNA genes were amplified via PCR for determining the prokaryotic and eukaryotic components of the biofilms respectively, using the primers listed in Table S1 (without fluorochromes). PCRs were performed using ~10 ng of DNA at the conditions specified in [31]. Amplified products were purified with the GeneAll Expin™ PCR SV kit (GeneAll Biotechnology Co., Seoul, Korea) and, if necessary, cloned into the pGEM-T easy Vector system following the manufacturer’s instructions (Promega, Vienna, Austria). Fragments were sequenced with Sanger chemistry in the 3130 Genetic Analyzer (Applied Biosystems, Foster City, CA, USA) using the BrightDye^®^ Terminator Cycle Sequencing Kit (Molecular Cloning Laboratories, Harbor Way, San Francisco, CA, USA). Electropherograms were visualised and, if needed, manually edited in the BioEdit software version 7 [32]. Sequences were taxonomically identified in the NCBI repository using the BLASTN algorithm [33] considering a minimum threshold >90% identity for bacteria and cyanobacteria and >95% for algae and fungi. The presence/absence of each taxon was then reported for each site of the grotto (tuff rock, dark fresco and light fresco).

#### 2.5.2. Automated Ribosomal Intergenic Spacer Analysis (ARISA) Capillary Electrophoresis and Community Analyses

The composition of the biofilm community in each sample was determined by ARISA. This is a PCR-based approach that allows a fast and cost-effective generation of whole-community fingerprints of bacterial, fungal and algal assemblages [34,35,36]. Total DNA was extracted from the biofilm samples collected in each tube using the procedure in [30]. PCRs were performed in a final volume of 25 μL using the primer sets listed in Table S1 at the following conditions: 10 × PCR buffer, 100 mM of dNTPs, 2.5 mM MgCl_2_, 0.5 μM of primers, 1 U of Taq polymerase (Qiagen, Hilden, Germany) and water to volume. The forward or reverse primers were labelled with different fluorescent probes as specified in Table S1. The PCR program consisted of an initial denaturation at 94 °C for 5 min and 35 cycles as follows: 30 s min of denaturation at 94 °C, 1 min of annealing at 55 °C, and 45 s of extension at 72 °C. A final extension of 6 min at 72 °C followed by cooling at 4 °C terminated the PCR program. Amplification success and quantification of reactions was determined by agarose gel electrophoresis (1% in 0.5 × TBE) with a 100 bp size standard. The sample fragments were then discriminated by using the 3130 Genetic Analyzer (Applied Biosystems, Thermo Fisher Scientific, Foster City, CA, USA) and the GeneTrace 500 plus LIZ as size standard (Carolina Biosystems, Ořech, Czech Republic), and subsequently analysed with Peak Scanner™ Software v1.0 (Thermo Fisher Scientific, Waltham, MA, USA). Peaks below the threshold of 2% total peak intensity were ignored. ARISA fragments were annotated in Microsoft Excel and assigned to bins of 3 bp (±1 bp). Different size fragments were considered as different species and transformed in presence/absence data.

All statistical analyses were performed in the R [37] package vegan [38] and plotted using ggplot2 [39]. The number of ARISA types for algae, bacteria, cyanobacteria and fungi was plotted in each substratum (tuff rock, dark and light frescoes). Non-metric multi-dimensional scaling (NMDS) was performed to visualise multivariate patterns in biofilm community structure based on the ARISA data generated from each biofilm sample. The analysis was performed using the metaMDS function, which performs multiple NMDS runs and retains the best solution, and the Jaccard’s distance [40]. To test whether the variance of the community was correlated with the environmental parameters recorded at each sampling site (Table 1), we first used the adonis function to assess which of the environmental parameters was statistically significant; then, those passing the test were analysed in a canonical correspondence analysis (CCA) framework in the same R package.

## 3. Results and Discussion

The Fornelle grotto was revealed to be a heterogeneous environment for both the abiotic and biotic parameters analysed. Temperature, light intensity and relative humidity were different across the three sampled environments of the cave (Table 1). The right side of the cave (the chapel), had the highest values of temperature and light intensity (27–28 °C and 500–680 lx), followed by the left side (~26 °C and 200–280 lx) and the end wall (~25 °C and 70 lx). Relative humidity was slightly higher in the left side (75%) than the end wall (73%) and right sides (70%).

**Figure 2:**
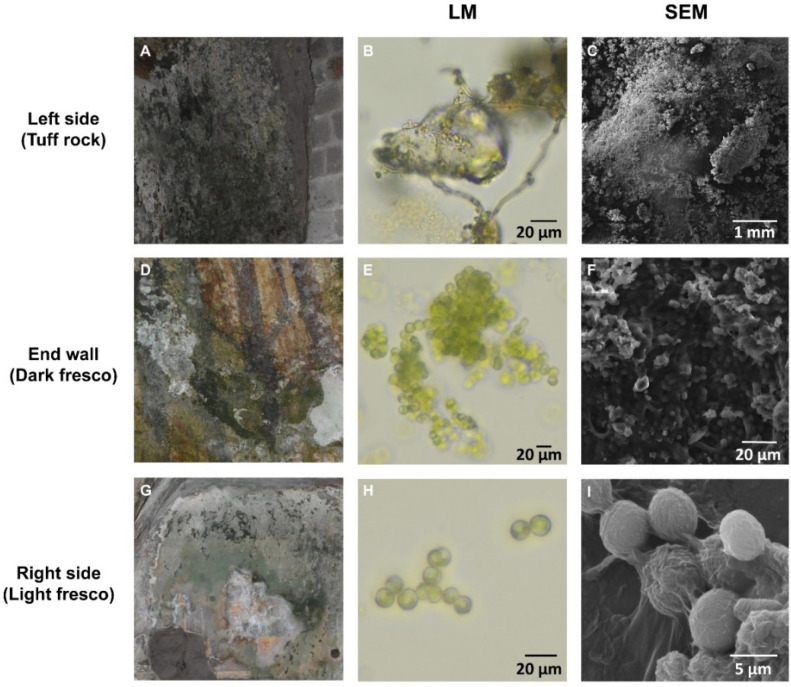
Photographic, light microscopy and scanning electron microscopy (SEM) images of biofilms sampled in the Fornelle cave. (**A**–**C**) tuff rock; (**D**–**F**) dark fresco; (**G**–**I**) light fresco.

Eubacteria, cyanobacteria, microfungi and green algae represented the great majority of observed microorganisms. Additional microorganisms, such as other algal groups, were only observed sporadically. Visual observations of the differently coloured biofilms revealed the presence of various microorganisms (Figure 2A,D,G), in accordance with other studies (e.g., [41,42]). On the left side of the cave, we observed mostly dark biofilms (intense browns, blacks and greens), suggestive of the presence of mostly fungal and bacterial communities (Figure 2A–C). The biofilms sampled on the end wall of the Fornelle cave formed patinas of different colours; the green ones were the most abundant, but we also observed white patinas with darker shades (Figure 2D). Observations at the optical microscope indicated the presence of abundant photoautotrophic microorganisms (Figure 2E) but also prokaryotic organisms were detected. Green patinas were also widely distributed on the surface of frescoes located in the right side of the cave (the chapel) (Figure 2G,H). SEM observations allowed us to understand the structure of biofilm on the various surfaces and to evaluate the type of interactions between microorganisms and substrates. Coccoid cells surrounded by a mucilaginous sheath were always present on the biofilms growing on pigments (Figure 2F,I). This association was also found on biofilms from the monastery of Santa Maria de Olearia, a limestone grotto at sea level on the coast near Maiori (Campania region, Italy), and the church of San Michele, a limestone grotto at 600 m above sea level, near Faicchio (Campania region, Italy) [41]. In Figure 2F, coccoid cells presumably belonging to green algae and cyanobacteria interacting with filamentous cells of bacteria and fungi were observed. On tuff rocks, the biofilm presented a packed structure formed by coccoid cells and fungal hyphae (Figure 2C).

X-ray diffraction analyses showed that the substratum of the samples from the chapel was mainly constituted by plaster, followed by calcite; all the other elements such as quartz, feldspar (an orthoclase, albite and partly anorthite), pyroxenes (mostly diopside), micas (biotite), and iron oxides (hematite) were found in small quantities (Table 2). Samples from the end wall and left side of the cave were mainly made of calcite, and presented low quantities of iron oxides and pyroxenes, and slightly different quantities of feldspars and plaster (Table 2).

We identified 47 taxa in the biofilm samples (Table 3) using Sanger sequencing from cultured samples. Specifically, we identified 8 bacterial, 18 cyanobacterial, 14 algal and 7 fungal taxa. Among bacteria, we found representatives of Actinobacteria (*Microbacterium* and *Micrococcus*), Bacteroidetes (*Bacteroides*), Gamma-proteobacteria (*Pseudomonas*) and Firmicutes *(Bacillus* and *Staphylococcus*). Some taxa were exclusive of one substratum (e.g., *Microbacterium* sp., *Pseudomonas* sp. and *Staphylococcus* sp.); others were present in two different substrata (e.g., *Bacillus megaterum* and *Bacillus mycoides*). The bacterial taxa observed have been also documented in other caves [41,43] as well as on mural paintings [44,45] and considered by some authors as the first colonizers of these environments [46,47].

The algal component of the biofilm mainly constituted green algae, and occurred in sites nearby the entrance of the cave lit by direct or indirect sunlight (Table 3). Most taxa belonged to Trebouxiophyceae, while all the others to Chlorophyceae, in accordance with other cave studies [48,49]. One of these species, *Bracteacoccus minor*, is a well-known source of damage on wall paintings in the Lascaux cave [50], but for the other species literature data are scarce and ours are among the first reports.

Coccoid cyanobacteria (*Aphanothece*, *Jaaginema*, *Prochlorococcus* and *Synechococcus*) were exclusively found on biofilms growing on the outermost, more lightened portions of the cave. Filamentous cyanobacteria were also found in the innermost part of the cave (Table 3), due to their capability of tolerating even extreme conditions of lighting and humidity [51,52,53]. These trends were also observed in a prehistoric limestone cave on Mount Carmel in Israel [54]. The cyanobacteria genera *Leptolyngbya*, *Microcoleus* and *Phormidium* detected here have been also found in other caves [55,56], while others (*Nodosilinea* and *Oculatella*) in archaeological sites [57,58] and also on monumental fountains (*Aphanothece* and *Pseudanabaena*) [59].

All fungal taxa identified here belonged to Ascomycota: two of them were exclusive to the light fresco substratum (*Cladosporium* sp. and *Fusarium verticilloides*), while the others were present on different substrata (Table 3). These taxa are well-known inhabitants of bare cave environments [60,61,62] as well as cave wall paintings [63,64] or cultural heritages in general [65]. Fungi were mostly found on tuff rock, followed by frescoes in dark and light conditions (Table 3).

**Figure 3:**
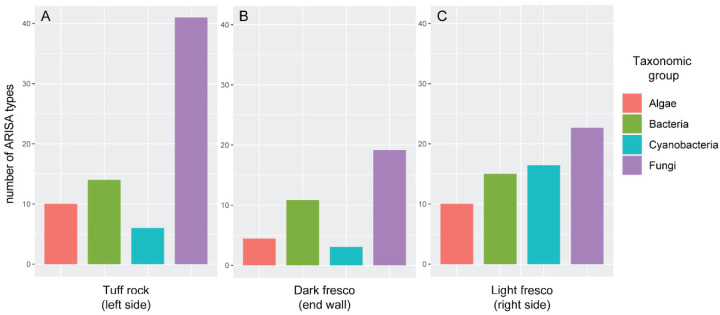
Automated ribosomal intergenic spacer analysis (ARISA) profiles of the microbial communities. (**A**) tuff rock (left side); (**B**) dark fresco (end wall); (**C**) light fresco (right side).

The ARISA analysis revealed that the number of ARISA types (putative different taxa) belonging to each taxonomic group considered was different across the three substrata analysed (Figure 3). The tuff rock substratum was largely dominated by fungi, followed by bacteria and algae in comparable numbers, and less than 10 cyanobacterial types (Figure 3A). The dark fresco community was rich in fungi and bacteria but poor in algae and cyanobacteria (Figure 3B). The light fresco community was the most homogeneous in terms of ARISA types among fungi, bacteria, cyanobacteria and algae, with comparable numbers of types (Figure 3B). Nonetheless, fungi presented the highest number of ARISA types, algae the lowest. In general, the fungal component accounted for the highest number of ARISA types in all substrata, with prevalence on tuff rock (Figure 3A). Cyanobacterial types were higher on dark fresco than on tuff rock and light fresco (Figure 3). Algal and bacterial types showed similar numbers in tuff rock and dark fresco substrata (Figure 3A,B). In general, the results of the ARISA are comparable, in terms of trends of occurrence of algae and cyanobacteria, with the ones obtained with the culture-approach. However, the number of fungal and bacterial taxa is greatly underrepresented in the latter approach, probably because most of the fungi and bacteria cannot be cultured with traditional methods. As a general trend, we observed a reduction in green algal biodiversity from the outermost to the deepest areas of the cave environment.

**Figure 4:**
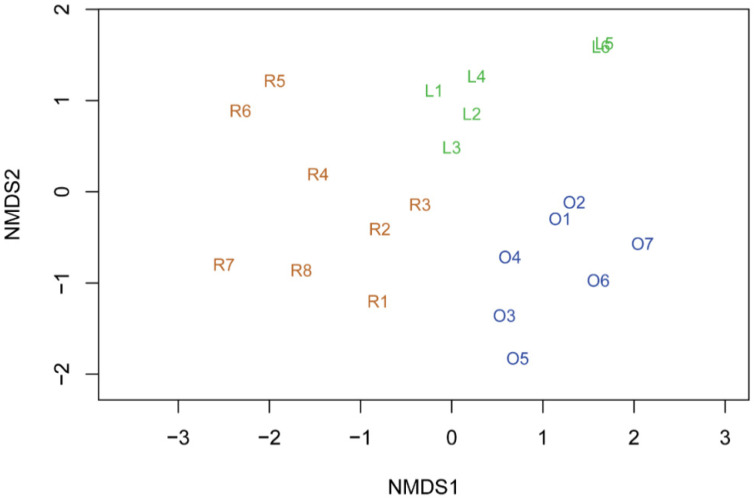
Non-metric multi-dimensional scaling (NMDS) plot built on ARISA profiles. Colours refer to different samples: brown = tuff rock; green = light fresco; blue = dark fresco.

The non-metric multi-dimensional scaling (NMDS) plot built on ARISA profiles allowed the identification of three groups (stress = 0.101), roughly corresponding to the three substrata (Figure 4). Samples from the tuff rock substratum are the most heterogeneous, followed by light and dark frescoes, the latter being quite homogeneous.

**Figure 5:**
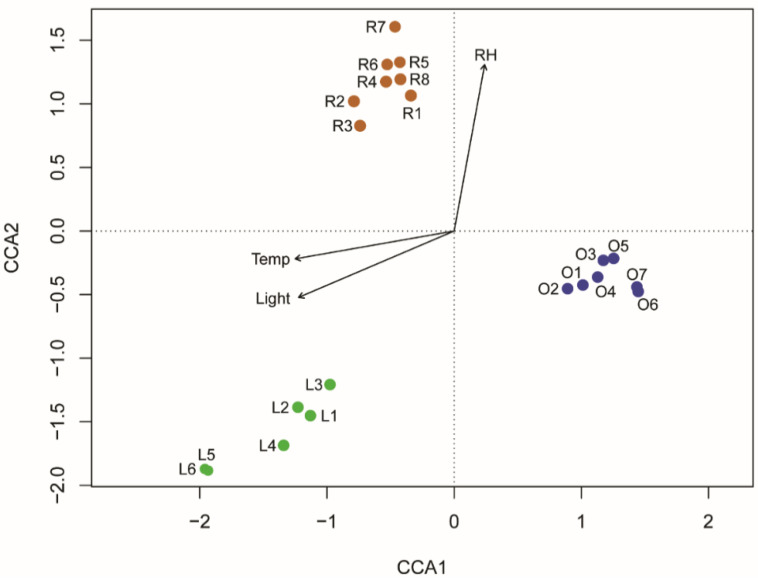
Canonical correspondence analysis (CCA) showing the correlation between biofilm communities and environmental parameters. For colour legend, refer to Figure 4. RH = relative humidity; Temp = Temperature (°C).

Temperature, light intensity and relative humidity registered at each sampling site were slightly positively correlated with the variance of biofilm community (temperature: r^2^ = 0.154; light intensity: r^2^ = 0.162; relative humidity: r^2^ = 0.165). These correlations were all significant (*p* < 0.001) and, accordingly, included in CCA analysis. All the environmental parameters had the same weight in the definition of the biofilm community. The CCA showed that the aforementioned environmental parameters explain the 27% of observed variation (inertia). The first axis (CCA1) explained 11.2% of the variation, while the second one (CCA2) 10.5%. Samples of light fresco (L1-L6) are slightly positively correlated with light intensity and temperature, whereas dark fresco samples are negatively correlated with these parameters (Figure 5).

## 4. Conclusions

The Mediterranean basin hosts many karstic caves, but only a limited number of these are accessible as show caves, which most often contain wall paintings (mostly dated to the Paleolithic and Neolithic). This study represents the first investigation on the characterization of the biofilm that colonises the different substrates at the Fornelle cave, a show cave harbouring Medieval paintings. It also constitutes a pilot study for the other votive caves in the archaeological site of Cales. As typical for caves, we found a decrease in the biodiversity of phototrophic organisms moving from the brightest parts of the cave to the more internal and dark ones. Conversely, the persistence of heterotrophic taxa has been confirmed in the darkest and most humid environments. We found different taxonomic compositions of the communities that inhabit the bare tuff walls of the cave and the frescoes in light and dark conditions. We also documented a positive correlation between light-requiring microbial communities (i.e., photoautotrophs), light intensity and temperature, and a negative correlation between these parameters and microbial communities growing in the dark (i.e., heterotrophs). These results highlighted that abiotic factors can shape the composition of a community even at small scale as in the case of a cave. The presence of different biofilms provided important information for the conservation of mural paintings.

## Figures and Tables

**Table 1:** Sampling points at Fornelle cave, with measures of corresponding environmental parameters on the tested samples.

Sample	Cave Side	Substratum	Temperature (°C)	Light Intensity (lx)	Relative Humidity, RH (%)
L1	right	Fresco (light)	27 ± 0.2	499 ± 0.1	70 ± 1
L2	right	Fresco (light)	27 ± 0.2	500 ± 0.2	71 ± 1
L3	right	Fresco (light)	27 ± 0.1	494 ± 0.3	70 ± 2
L4	right	Fresco (light)	27 ± 0.1	497 ± 0.1	68 ± 1
L5	right	Fresco (light)	28 ± 0.1	680 ± 0.1	68 ± 1
L6	right	Fresco (light)	28 ± 0.2	678 ± 0.1	69 ± 1
O1	end wall	Fresco (shadow)	25 ± 0.1	68 ± 0.1	73 ± 1
O2	end wall	Fresco (shadow)	25 ± 0.1	68 ± 0.2	73 ± 1
O3	end wall	Fresco (shadow)	25 ± 0.3	70 ± 0.2	73 ± 1
O4	end wall	Fresco (shadow)	25 ± 0.2	69 ± 0.1	72 ± 1
O5	end wall	Fresco (shadow)	25 ± 0.1	67 ± 0.3	73 ± 1
O6	end wall	Fresco (shadow)	25 ± 0.2	70 ± 0.2	73 ± 1
O7	end wall	Fresco (shadow)	25 ± 0.1	70 ± 0.1	73 ± 1
R1	left	Tuff rock	26 ± 0.1	280 ± 0.3	75 ± 1
R2	left	Tuff rock	26 ± 0.1	278 ± 0.3	75 ± 2
R3	left	Tuff rock	26 ± 0.2	280 ± 0.2	75 ± 1
R4	left	Tuff rock	26 ± 0.1	200 ± 0.2	75 ± 1
R5	left	Tuff rock	26 ± 0.2	200 ± 0.2	75 ± 1
R6	left	Tuff rock	26 ± 0.1	198 ± 0.1	75 ± 1
R7	left	Tuff rock	26.1 ± 0.1	200 ± 0.1	75 ± 1
R8	left	Tuff rock	26.2 ± 0.1	200 ± 0.1	75 ± 1

**Table 2:** Mineral composition of substrata.

	Light Fresco	Dark Fresco	Tuff (Bare Rock)
**Calcite**	++	+++	+++
**Feldspars**	+	+	++
**Iron oxides**	+	+	+
**Mica**	+	+	+
**Plaster**	+++	++	+
**Pyroxenes**	+	+	+
**Quartz**	+	+	+

+++ = very abundant; ++ = abundant, + = less abundant, + = traces.

**Table 3:** Taxa identified in the Calvi Risorta grotto with Sanger sequencing.

Taxa	Dark Fresco	Light Fresco	Tuff Rock
**Bacteria**			
*Bacillus megaterum*	-	+	+
*Bacillus mycoides*	-	+	+
*Bacillus* sp.	-	+	+
*Bacteroides* sp.	-	-	+
*Microbacterium* sp.	+	-	-
*Micrococcus* sp.	-	-	+
*Pseudomonas* sp.	-	+	-
*Staphylococcus* sp.	-	+	-
**Cyanobacteria**			
*Aphanothece naegelii*	-	+	-
*Halospirulina tapeticola*	-	-	+
*Jaaginema* sp.	-	+	-
*Leptolyngbya africana*	+	-	-
*Leptolyngbya faveolarum*	+	+	-
*Leptolyngbya norvegica*	+	+	-
*Microcoleus* sp.	-	-	+
*Nodosilinea bijugata*	+	-	-
*Nodosilinea* cf. *nodulosa*	+	-	-
*Nodosilinea* sp.	+	-	-
*Oculatella subterranean*	-	+	-
*Oculatella ucrainica*	-	+	-
*Oscillatoria angusta*	-	+	-
*Phormidium* sp.	-	+	-
*Prochlorococcus* sp.	-	+	-
*Pseudanabaena limnetica*	-	-	-
*Spirulina* sp.	-	-	+
*Synechococcus* sp.	-	+	-
**Algae**			
*Auxenochlorella protothecoides*	-	+	-
*Bracteacoccus xerophilus*	-	+	-
*Chlamydomonas chlamydogama*	-	+	-
*Chlorella sorokiniana*	+	-	+
*Chlorella* sp.	-	+	-
*Chlorella thermophile*	-	+	-
*Chlorella vulgaris*	-	+	+
*Chloroidium saccharophilum*	-	+	+
*Dictyosphaerium ehrenbergianum*	-	+	-
*Didymogenes sphaerica*	-	+	+
*Eremochloris sphaerica*	-	-	+
*Marvania coccoides*	-	+	-
*Micractinium reisseri*	-	+	-
*Neochloris aquatica*	-	+	-
**Fungi**			
*Alternaria* sp.	+	-	+
*Aspergillus* sp.	+	-	+
*Cladosporium* sp.	-	+	-
*Colletotrichum* sp.	-	-	+
*Fusarium verticilloides*	-	+	-
*Penicillium* sp.	+	-	+
*Pleosporales* sp.	-	-	+

## Data Availability

The data presented in this study are available in the article and as online supplementary material.

## Supplementary Materials

The following are available online at https://www.mdpi.com/article/10.3390/ijerph18158048/s1: Table S1: List of primers utilised for ARISA.
