# Supplementary material for: Characterisation of Environmental Biofilms Colonising Wall Paintings of the Fornelle Cave in the Archaeological Site of Cales
Source: Int J Environ Res Public Health. 2021 Jul 29;18(15):8048. doi: 10.3390/ijerph18158048 (PMC8345495; doi:10.3390/ijerph18158048)
Supplement: Supplementary file 1 [file ijerph-18-08048-s001.zip › ijerph-1252506-supplementary.pdf]

Table S1. List of primers utilised for ARISA.

Primer	Sequence 5'→3'	Target region	Target group	Reference
**16S1515**	AGT CGT AAC AAG GTA GCC GTA CC	16S-23S ITS	Cyanobacteria	Cardinale et al., 2004 (modified)
**B23S-6FAM (R)**	CTT CGC CTC TGT GTG CCT AGG T	16S-23S ITS	Cyanobacteria	Lepère et al., 2000
**ITS (F)**	GTC GTA ACA AGG TAG CCG TA	16S–23S ITS	Eubacteria	Cardinale et al., 2004
**ITS(R)eub-HEX**	GCC AAG GCA TCC ACC	16S–23S ITS	Eubacteria	Cardinale et al., 2004
**ARALG18S (F)-PET**	GAA CTT GTC TAA ACC TTA TCA T	ITS1-5.8S-ITS2	Algae	Fechner et al., 2010
**ITS4 (R)**	TCC TCC GCT TAT TGA TAT GC	ITS1-5.8S-ITS2	Algae	White et al., 1990
**2234C-NED (F)**	GTT TCC GTA GGT GAA CCT GC	ITS1-5.8S-ITS2	Fungi	Ranjard et al., 2001
**3126T (R)**	ATA TGC TTA AGT TCA GCG GGT	ITS1-5.8S-ITS2	Fungi	Ranjard et al., 2001
